# Unlocking the forest: An ethnographic evaluation of Forest Schools on developmental outcomes for 3-year-olds unaccustomed to woodland spaces

**DOI:** 10.12688/wellcomeopenres.22851.1

**Published:** 2024-09-09

**Authors:** Anna Cronin de Chavez, Amanda L. Seims, Josie Dickerson, Nimarta Dharni, Rosemary R. C. McEachan

**Affiliations:** 1 London School of Hygiene and Tropical Medicine, Faculty of Epidemiology and Population Health, London, England, UK; 2 Bradford Institute for Health Research (on behalf of the Better Start Bradford Innovation Hub), Bradford Teaching Hospitals Foundation Trust, Bradford, BD9 6RJ, UK; 3 Institute of Applied Health Research, University of Birmingham, Birmingham, UK

**Keywords:** Child development, Early childhood experiences, Early years foundation stage, Nature-based play, Outdoor learning

## Abstract

**Background and purpose:**

Early years experiences shape a child’s physical, cognitive and emotional development. Spending time in greenspaces offers benefits for children’s development, but access and use can be limited in urban settings. There is increasing interest in the health and developmental benefits of Forest Schools for primary-aged children, but little is known about the benefits for pre-school children. This study aims to identify these and explore the processes and activities associated with a Forest School intervention for early years children that may influence outcomes.

**Methods:**

This paper reports on an ethnographic approach involving 65 hours of observations with two cohorts of 10 3-year-olds attending 11 weekly Forest School sessions in an urban setting. The children attending had little or no previous experience of natural spaces. 14 in-depth interviews were conducted with parents, and nursery and Forest School staff. The data were analysed using thematic analysis, and outcomes were identified using the Early Years Foundation Stage Statutory Framework.

**Results:**

Despite logistical challenges, the intervention benefitted age-specific health and development outcomes, particularly personal, socio- and emotional development, verbal communication, and mathematics. Unexpected benefits were observed among nursery staff and parents attending Forest School.

**Conclusion:**

Forest Schools are a promising and feasible method to improve nature connectedness and development in children aged 3 years and support school readiness. The maintenance and protection of urban woodland spaces are paramount to facilitate this.

## Plain language summary

Early years experiences affect children’s physical, cognitive and emotional development. Spending time in green spaces offers benefits for children’s development, but access and use can be limited in urban settings. Forest schools have become increasingly popular for nurseries and kindergartens. The benefits of Forest Schools for primary-aged children have been explored, but little is known about the benefits for pre-school children. A Forest School programme in an urban area of Bradford was evaluated to understand children’s experiences and benefits.

Dr Chavez observed 20 3-year-olds attending 11 weekly Forest School sessions. Observations totalled 65 hours. Dr Chavez also interviewed 14 parents, and nursery and Forest School staff. Dr Chavez and Dr Dharni used the Early Years Foundation Stage Statutory Framework and looked for patterns in the interview data to understand the benefits children had gained.

The results showed that Forest School for this age group had benefits for age-specific health and development outcomes, particularly personal, socio- and emotional development, verbal communication, and mathematics.

Forest School is a promising and feasible method to improve connection to nature and development in children aged 3 years, and help prepare them for school. Forest School can support very young children to enjoy and be safe in natural environments. It can help children cope with change from being mainly at home with family and carers, to being at school and in their neighbourhood.

## Introduction

The first three years of life are critical for a child’s physical, cognitive and emotional development. Negative experiences during this vital period can have a lifelong impact on a child’s health, wellbeing and life chances ^1, 2^. Only 65.2% of children in England achieved a good level of development (indicating school readiness) at the end of reception in 2022 ^3^. Attending good quality Early Childhood Education and Care has been shown to improve several early years developmental outcomes for children ^4^, and there is some evidence that access to green spaces and nature can contribute to children’s physical and psychological health, and development ^5– 8^.

However, children’s access to, and use of green space in urban areas is limited. Previous research in Bradford highlights that parents of preschool children recognise the benefits of playing in green spaces and interacting with nature, however structural barriers within the built-environment, parents’ lack of knowledge of suitable spaces to play, and parents’ confidence in managing children’s behaviour and keeping them safe restricts children’s opportunities ^9^. Furthermore, South Asian children’s play in green space is limited through typically residing in areas with less green space, and lower quality green space than White British children ^10^.

Globally the Covid-19 pandemic resulted in an increased awareness of the benefits of nature and green spaces. Over the pandemic there were several calls to provide more outdoor learning opportunities for children at school during the Covid-19 pandemic to reduce risks of contagion ^11^, and to mitigate against the psychosocial damage children may have faced during lockdown ^12^. In the post-pandemic era, we are now seeing some of the negative developmental impacts for those born, or who were very young, during the different periods of lockdown and reduced social interaction in the UK ^13^. There is also increasing concern regarding the climate emergency, and emerging evidence that a connection with nature helps develop positive conservation behaviours ^14^.

Forest School is one potential intervention that could support children’s health and development in the critical early years through increased access to the benefits of nature. A ‘quality Forest School’ is defined by the UK Forest School Association as a delivery that holds 6 specific principles that in summary include: 1) a long-term adaptive approach; 2) takes place in a woodland or natural space; 3) uses a range of learner-centred processes; 4) promotes holistic personal development; 5) supports risk taking; and 6) the Forest School is run by qualified Forest School practitioners ^15^. The first Forest School in the UK was in 1993 ^15^, with foundations in the educational and philosophical movements of the 1800’s and 1900’s and influenced by Froebel, Steiner, Montessori and scouting and guiding movements.

Forest Schools have been shown to benefit children’s cognitive function, motor coordination and balance, connectedness to nature, and health and well-being outcomes ^16^ and improved academic attainment ^17^. Other research has suggested that benefits may include a positive impact on confidence, social skills, language and communication, motivation and concentration, physical skills and knowledge and understanding ^18, 19^. Forest Schools may also help with children transitioning from home to school ^20^. However, most Forest School provision and evaluation thereof, has been for children of primary school age (4 years+), and there is limited evidence on potential health and developmental benefits of Forest Schools or nature play for pre-school children ^16^.

The aim of this evaluation was to explore the potential health and developmental outcomes resulting from a pilot intervention of a Forest School programme for 3-year-olds attending nurseries in urban settings. It also aimed to understand the key structured and non-structured processes and activities alongside outcomes, to hopefully inform future forest school programmes for very young children. 

## Methods

### Project context and setting

**Figure 1.**
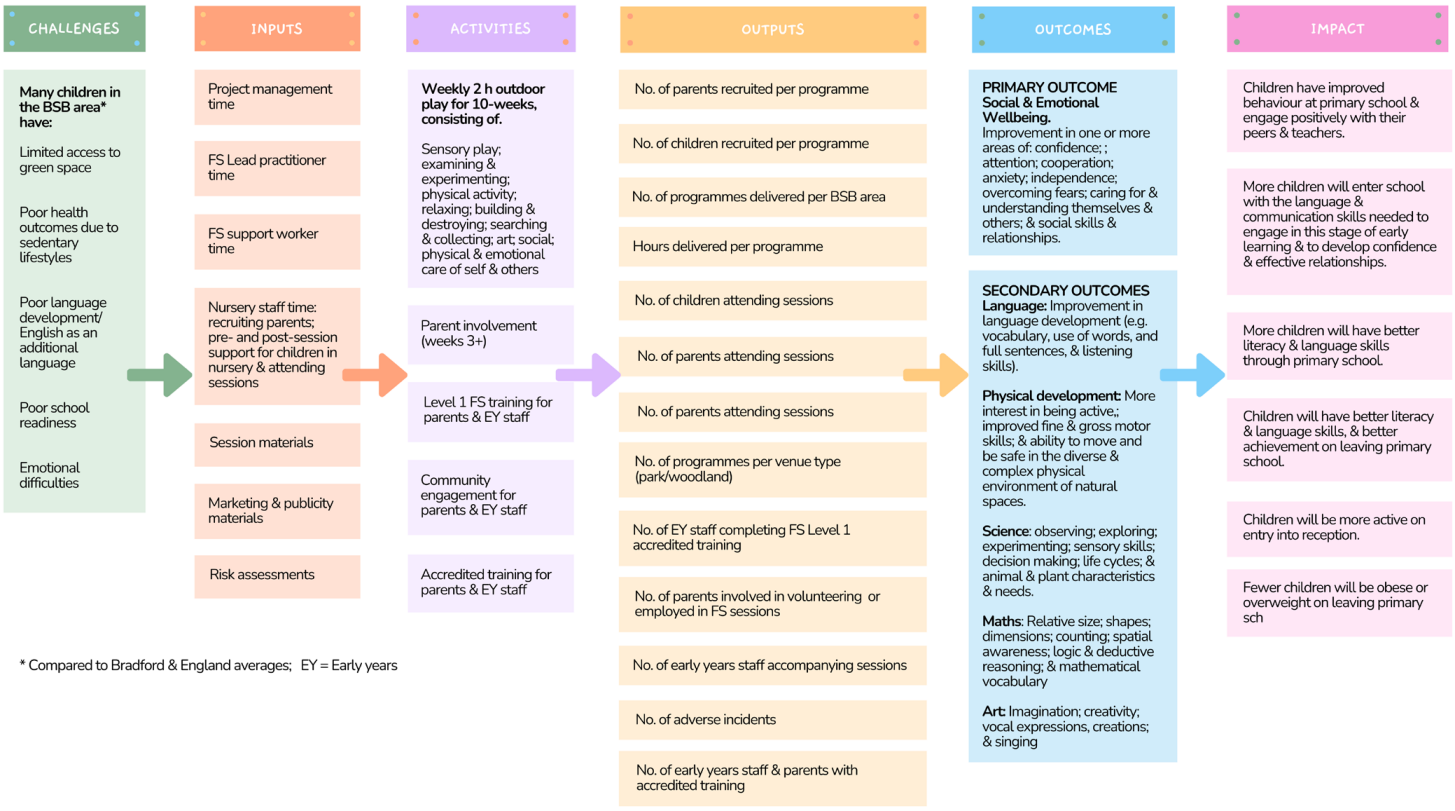
Forest school intervention logic model (created by Amanda Seims).

Only 62.3% of children in Bradford achieved a good level of early years development, indicating school readiness, at the end of reception in 2022; nearly 3% lower than across the UK ^3^. Bradford is a large, urban multicultural city in the North of England, UK. With a population of over 546,000 it is the 7 ^th^ largest metropolitan district in England and Wales ^21^; 26% of the population are of Pakistani origin ^22^. Fourteen of the districts’ 30 wards are amongst the 10% most deprived in England and Wales ^23^. In 2015, Better Start Bradford was funded by the National Lottery Community fund to implement a range of early years interventions, to improve the socio-emotional, language and communication development, and nutrition of children aged 0–3. The Better Start Bradford areas are ethnically diverse, highly deprived and have a large proportion of children who have poor early years outcomes ^24^. As a part of the Better Start Bradford programme, the Better Start Bradford Innovation Hub was established to evaluate the impact of all the interventions as delivered in usual practice. One of the interventions selected for implementation and evaluation was a Forest Schools pilot for pre-school children. Better Start Bradford led the development of a logic model for this pilot with the involvement of parents and other stakeholders ( Figure 1)

### The intervention

A local Forest School company was commissioned to provide the service for 3-year-olds attending state-funded nurseries in areas covered by the Better Start Bradford programme.

The intervention involved nature play in local green spaces such as in local parks and woods, led by trained Forest School leaders and accompanied by nursery staff and sometimes parents. The Forest School provider delivered the programme in four nursery schools over the initial year of delivery (2017–2018), engaging 10 children per nursery. Two level 3 (qualification for Forest School leaders, higher than level 1 for Forest School assistants) Forest School qualified staff, both with previous experience working with this age group, ran each session, accompanied by two nursery staff. Parents were invited to accompany the group and were also offered places on a three-day level 1 Forest School training course, with childcare provision.

**Figure 2.**
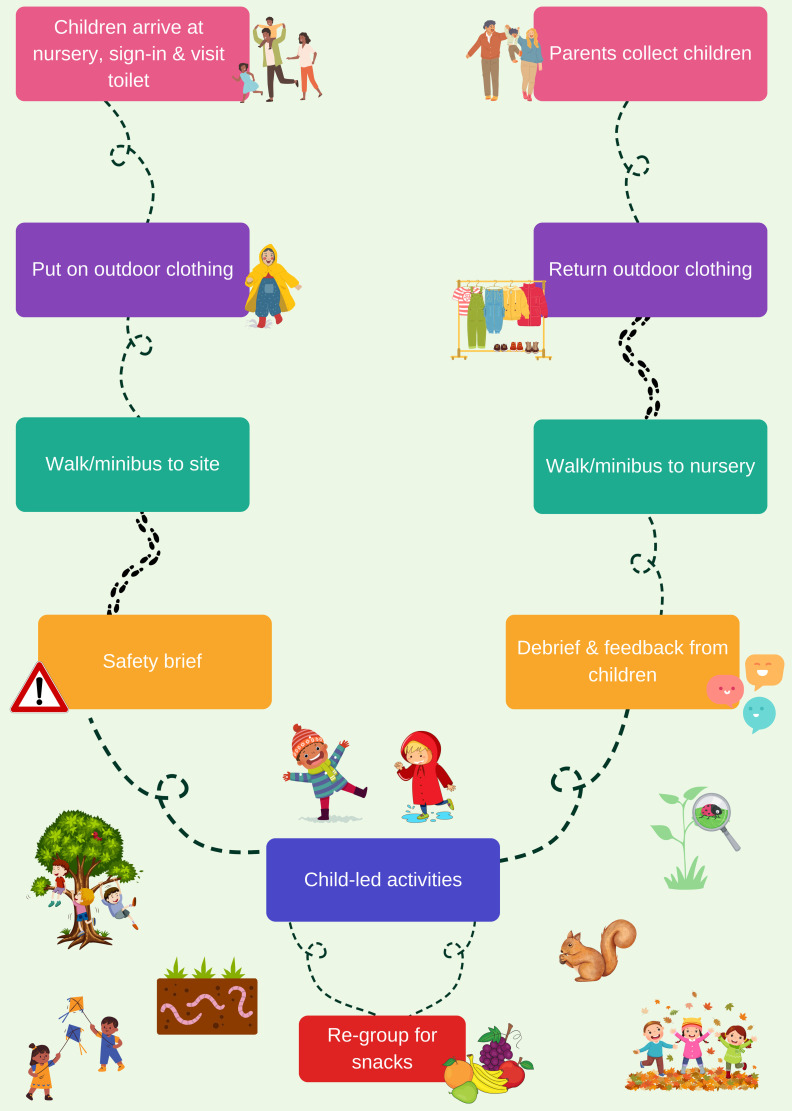
Forest School session routine (created by Amanda Seims).

The first session for each nursery school was held in the nursery yard, four were run in a local park and for the last six, the children and staff were transported by minibus to a woodland, 20 minutes’ drive from the nursery. Two nursery staff always accompanied the children. The Forest School leaders would arrive at the nursery to help get the children ready to go outside. Good quality waterproofs and warm weather gear had been funded by the project and nurseries were allowed to keep the clothing for use outside the sessions. The Forest School leaders brought equipment and first aid with them to the Forest School site and the nursery staff brought refreshments and hygiene materials. Risk assessments were completed for all sites in advance of sessions, and repeated throughout the programme depending on the weather, changes to the site and temperament of the children. Some open sessions for local families were also run on Saturday mornings for families but were not attended by the evaluator because consent would not have been possible for open sessions. Whilst Forest School principles emphasise activities being highly child-led, there was a very basic structure to each session, tailored for their age ( Figure 2).

### Design

A qualitative approach including ethnography and interviews was used to identify potential outcomes associated with the Forest School programme. 

Existing methods for evaluating Forest Schools for older children have included satisfaction questionnaires for staff and children, staff assessment of child’s experience, and draw and write techniques ^25, 26^. These methods have limited suitability for young children with limited written and spoken abilities. However, children of all ages have a right to participate in evaluation ^27^, and have their views considered when designing services they are to receive, such as a Forest School service ^28^.

For this evaluation, understanding the children's experience through ethnographic methods were chosen because of the potential for the children to gain trust with the evaluator in their own time and to a greater extent carry on as normal while their experiences, interests, and relationships with staff and the environment were observed and recorded. Spending time with the children while they carried out their normal activities allowed for informal conversations, and observation of social interaction and non-verbal communication such as facial expressions and body language, laughter, crying and reactions.

Ethnography is a commonly used method to gain insight into difficult to access groups that are not well understood by outsiders, in this case, 3-year-olds. It aims to see the world from the perspective of those studied, and explores meaning, social interactions and relationships within a chosen group ^29^. Ethnographic methods also allow for the triangulation of various data sources and can provide an understanding of an intervention and how it is delivered and received, creating data on how, why and when positive and negative outcomes occurred. 

Any evaluator conducting participant observation and in-depth interviews will inevitably approach the evaluation with their own set of personal beliefs, experiences and preconceptions so it is important for them to be aware of how their own background and experience may bias the data collected. The evaluator for this project (AC) had a background in applied qualitative research with ethnic minorities and experience working with children in forest settings – indigenous children living in a Panamanian rainforest and UK children learning about orienteering; a more structured use of UK green space. AC also had experience of qualitative interviews with parents on use of green spaces for families with children aged 0–3 in the same area of the city but not involved in this intervention ^9^. Although AC had fieldwork experience with participants from similar backgrounds in natural environments, she was not from their ethnic background, nor was it her first time in woods as it was for some of the participants so her perspectives may have been different. Also being an adult, she could not necessarily understand the perspective of a 3-year-old, but that is why the method of ethnography was chosen as it attempts to understand their perspective in a more child friendly way than doing formal interviews.

### Ethical considerations and data protection

This evaluation was deemed by the Health Research Authority to be service evaluation (HRA decision 60/88/81), not research, so ethics approval was not required. However, the researchers deemed it important to apply principles of good research practice and governance. Parents and staff were given participant information sheets (available on Harvard dataverse – see extended data section) for the observations and interviews, with an opt-out choice for observations and signed consent forms for interviews (available on Harvard dataverse – see extended data section). Children were verbally informed that they would be observed as part of the evaluation. Pseudonyms were allocated to each participant to protect their anonymity and for the purpose of linking data from different methods. Audio data were stored in a password protected file on an encrypted online server (Bradford Institute for Health secure internal drive) and written data were stored in a locked filing cabinet. Anonymised data were only accessible by the core evaluation team. A copy of the evaluator’s Disclosure and Barring Service (DBS) certificate was taken by each participating nursery school.

### Participants

The Forest School company built the initial rapport with the schools, parents and children and then they introduced AC to everyone as someone they trusted and were working with. At each of the two nurseries delivering the Forest School programme, ten 3-year-old children (n=20), their parents accompanying them (n=20), two nursery staff (n=4) and the Forest School leaders (n=2) were included in the observation element of the evaluation. The staff in one nursery already had some exposure to Forest Schools, having had the use of a natural outdoor play area, and were very keen on engaging with Forest Schools, and the other was newer to the activity. 

The demographics of the participants were representative of the Better Start areas where the intervention was being delivered, where 88% are from an ethnic minority, the majority of whom are of Pakistani heritage (61%), and most families (84%) live in the lowest decile of the Index of Multiple Deprivation.

### Data collection

***Participant observation.*** The nursery and Forest School staff were informed of the evaluation prior to the commencement of the Forest School programme, and agreed the processes and activities. Prior to attending a taster session, parents were provided with an information sheet which explained the observation methods used for the ethnographic aspect of the evaluation, how the data would be anonymised, and the procedures to opt-out if they did not consent to taking part. The children, nursery staff, Forest School staff and parents attending the sessions were observed through their experience over 11 sessions per group. This was over the period September 2018 to January 2019 and involved a total of 65 hours of participant observation. AC explained she was there to observe the project and write some notes, and informed the participants they could ask questions about it at any point in time during the fieldwork. She explained who she was working for (Born in Bradford at Bradford Institute for Health Research), her previous experience in woodland environments and interest in nature to the adults. With the children, who were too young for more formal explanations, she demonstrated interest in a nature environment through an interest in the Forest School activities or talked about past experiences in woodlands (such as what wildlife she'd seen before). Each session was observed during orientation and registration, getting ready to go, travel to and from the site, the session itself, time back in nursery getting ready to go home and parents picking up children. AC observed social interaction and non-verbal communication such as facial expressions and body language, laughter, crying and reactions, and participated through engaging in informal conversations. AC also had access to documents such as tender documents, project plans, logic models, meeting minutes and consultation surveys of practitioners and parents. AC sat in on planning and quarterly review meetings between January 2018 and March 2019 but had no say in decision making. The level of participant observation was moderate, i.e. AC did not lead or deliver any activities, but did have a limited degree of interaction and participation which was needed for the children and nursery staff to feel at ease in her company to carry on as normally as possible. As the ethos of Forest School is for everyone to participate in something, albeit an activity of their choice, it would have been against the spirit of the activity to not participate at all.

Notes of observations and conversations, activities, themes etc. were made throughout the sessions. Timing of observations was noted, and meanings or assumptions were inferred in notes and the context were described ^30^. Verbatim quotes were rarely possible, but to maximise recall all notes were written up immediately after the sessions finished.

### Parent and staff interviews

The evaluator approached parents at the time of nursery drop-off once the Forest School sessions had finished, and recruited them using convenience sampling. Purposive sampling was used to recruit nursery staff from those who regularly attended the forest school sessions. Four parents from each site (n=8), the nursery staff (n=4) and Forest School leaders (n=2) consented to participate, and took part in a 30–90 minute interview in a private room in the school when convenient. A semi-structured interview approach was used, with the topic guides (available on Harvard dataverse – see extended data section) facilitating discussions around their experiences of the Forest School programme and their observations of outcomes among the children.

A digital recorder was used for interviews where permission was given, and then transcribed. Where permission to record wasn't given, shorthand notes were taken during the interview and written up immediately afterwards.

### Analysis

Manual coding of field-notes was conducted throughout to discover lines of enquiry and focus as the fieldwork went on ^31^. Thematic analysis was used to analyse the observation and interview data, which was partially double coded by a second evaluator (ND). Results throughout the analysis were discussed with the evaluation team RM, JD and ND. To identify the potential positive or negative impacts of forest schools on 3 years olds’ development, we utilised the Early Years Foundation Stage Statutory Framework (EYFS) ^32^. This framework was chosen because it is central to child development assessments in early years settings, and so our results would be of significance to those working in this setting. It includes seven primary areas of development that are used to assess a child’s readiness to learn in this first year of primary school (aged 4–5 years old) in the UK ^32^. The seven areas of development are: 1) personal, social and emotional development; 2) communication and language; 3) physical development; 4) literacy; 5) mathematics; 6) understanding the world; and 7) expressive arts and design.

## Results

All 20 families were recruited to the intervention, with 20 children (10 from each nursery group). All children were 3 years of age, with 13 girls and 7 boys. All children could speak English to some degree, some were still learning English having been used to speaking a different language at home. The families came from West African, South Asian, Eastern European, White British, and mixed ethnic backgrounds.

**Figure 3.**
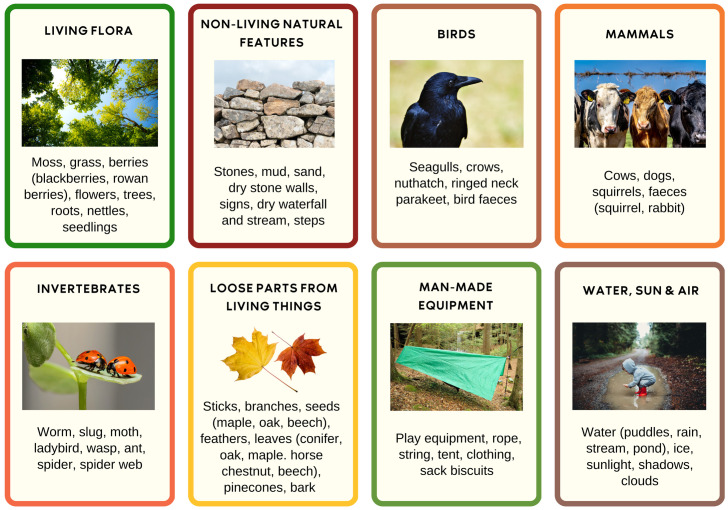
Natural materials and living things children used or watched for Forest School play (60+ items) (created by Amanda Seims).

One of the theories as to why Forest Schools may benefit children’s health and development is that a natural environment provides a large quantity of flexible environments and materials to provide multiple opportunities and experiences. The child-led activities resulted in children finding and using the below natural materials, plants and animals ( Figure 3).

**Figure 4.**
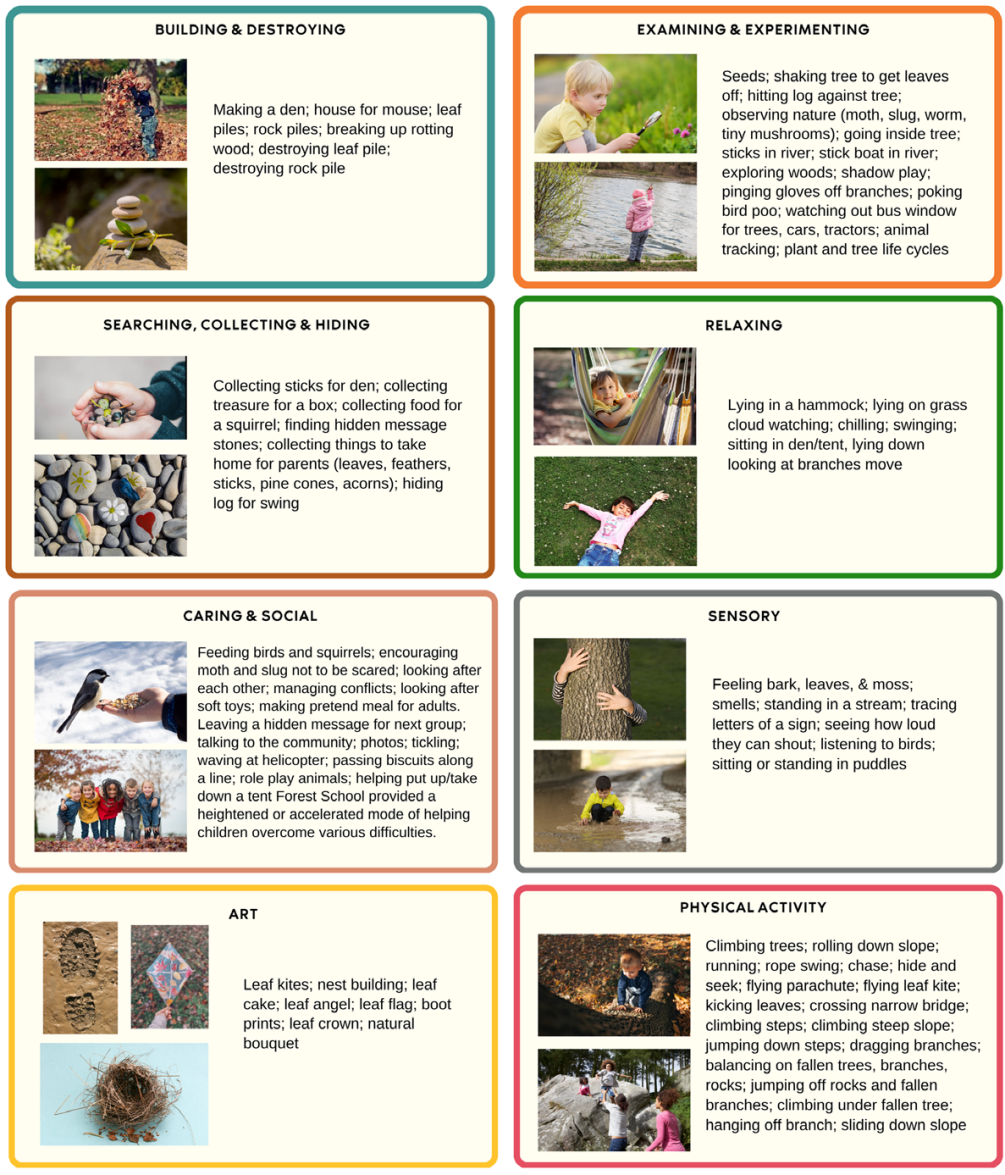
Overview of child-led Forest School activities children participated in (created by Amanda Seims).

The types of activities they developed or got involved in are listed in Figure 4. Again, the diversity of materials available just in a park and one limited section of woodland was quite extensive, allowing for multiple opportunities for creating activities. Over 80 different types of activities were recorded which would support the case for the diverse opportunities provided by natural environments.

This evaluation considered the processes and activities involved in forest schools for 3-year-olds and the potential impact across the seven EYFS primary domains. The findings suggest that Forest School provided a heightened or accelerated mode of helping children overcome various physical, psychological and cognitive challenges that impacted on the individual strengths and difficulties of each child. There appeared to be particular benefits for EYFS domains of personal, social and emotional development, with benefits also apparent for communication and languages; physical development; literacy; mathematics; understanding the world; and expressive arts and design. Examples are presented below.

### EYFS 1 Personal, social and emotional development

A positive impact on social and emotional wellbeing appeared to be a major outcome. Existing social and emotional challenges for several children included the transition to nursery and being separated from parents, difficult home situations, inexperience of group work, and the addition of being in the unfamiliar natural environment of the Forest School setting. There were examples where the children appeared to overcome multiple challenges and nursery staff and parents noted an improved confidence and reduced anxiety in their children. Examples are given below.

**Making relationships:** Starting nursery meant not just getting to know their nursery teachers, but also the other children. Lina, who regularly asked for her mum and could not participate in group activities, often took most of the session to become involved with the group, but would eventually start smiling and talking. Despite the appearance that she wasn’t participating, her mum reported her saying the Forest School made her feel like the whole group were her friends. Being in a smaller group, and having opportunities to cooperate and think of each other, mediated by adults, may have helped the group feel friendship and identity on a group level. There were constant opportunities for adults and children to cooperate, as almost a requirement to stay safe. The activities also required group cooperation through waiting in turn, such as to be led across the bridge over the stream, and helping put the log swing together and taking it down again. Popular activities inevitably required children to share materials and spaces, encouraging the children to interact and think of each other and adults supported them with this.

**Self-confidence and self-awareness:** Building confidence was apparent with the Forest School child-led activities, where children chose activities both on an individual and group level. One example is when the children discovered a dried-up stream bed, they chose to run along it and a small, dried-up waterfall, which they decided to climb. The Forest School and school and nursery staff supported their choice by mitigating any risks, placing themselves to stop children falling or running off. Some individual activities turned into group ones, such as Maira making her own pile of leaves - when the others saw, they joined in and swept up leaves to make a giant pile, which they then jointly decided they would destroy. Children also led adults, giving them instructions such as climbing under a tree or telling them they should stay with a group. There were however, some limits to child-led activities in this setting with this age group, such as safety, and children not accustomed to leading their own activities. Some children expected some reassurance in what they were doing: “ *They're waiting aren't they for somebody, yeah, somebody to say 'yeah, that's OK'.”* (School A Nursery staff (NS) 1)

Nursery staff and parents believed improving confidence was a major outcome for the Forest School:

*“Yasmin obviously her confidence has like absolutely rocketed, before I think she was quite nervous to come, she was excited but sort of nervous, and nervous to try things, now when she’s outdoors she’s like really confident on like the climbing equipment, and independence again, I think it definitely encourages independence and confidence more out of anything else, yeah.”* (School B, NS2)
*“But the most important thing is, he was scared of ducks, that is, he's not scared of ducks anymore, it's amazing. … now when we go there, “Oh ducks”, and taking stuff and throwing it and playing with the ducks, he's more joyful about it.”* (Zak’s dad)

Maira did not want to participate in many adventurous activities at first - she preferred to study things alone, a feather let go repeatedly to fall, study how the trees waved in the wind, scratch marks on sticks, how big they were, their leaves. Her mother said she was born premature, and they may have been quite protective of her, which they felt might explain why she wasn’t as adventurous as her older sister. During one session in the woods, Maira found a branch, two or three times the height of herself. Happily hauling it through the leaves around the woods, she declared out loud to everyone “ *I’m big and strong”.* This showed a positive change in her confidence and self-image.

There were several instances where children overcame fears during the session, being listened to, persuaded and supported to do things they perceived as scary. Zahra was anxious about going to new environments such as the park and while we were walking up to the entrance of the woods, became anxious there would be wild animals in there. This was not a completely illogical anxiety, given her knowledge of woods may have been gained thus far from stories in popular children’s books such as *The Gruffalo*, and *One Snowy Night*. With support from nursery and Forest School staff, she overcame these anxieties and enjoyed being somewhere new.

Some of the children took on leadership roles themselves. For example, one child who didn't like to be involved with group activities was also quite adventurous, and other children saw and liked the activities she had discovered by herself and joined her. Another girl stepped in to encourage her classmate to put on the waterproof clothing when the girl was resisting the instructions of adults. The children also led the adults, telling them to stay with the group or it was time to go, and teaching their parents what they have learnt in Forest School:

*“When we go outside, he'll touch a tree and he'll say, ’Oh, Mummy, that is very hard, you touch that tree and tell me if it's soft or hard’, so things like that. When I look at him I know, you can tell, he is leading, he wasn't in the lead, like that habit before, you know, to lead the things, but now I can see that in him, developing, he’s leading us, because he knows, because we just say, ‘Oh, we don't know if it's hard or not’, he goes, ‘Come on, I'll show you what it is', so as if he knows everything when we go outside." (Rafi’s mum).*

**Managing feelings and behaviour:** Children had opportunities to manage their feelings such as the trauma Rafi was experiencing at home while his parents were going through a divorce. The Forest School staff noted his quite severe mood swings during the sessions, going from happy, carefree running around, to being withdrawn, unresponsive and unwilling to even walk. His mother was sure the Forest School had helped him during this difficult period as a direct result of the natural environment and the connection the Forest School staff had made with him. Her occasional attendance had also helped her avoid depression:

*“Yeah, he's very bright, but because of all these circumstances for me he's seen at home, he goes a bit down sometimes, because sometimes I'll be very tearful at home and when he sees me, he gets upset as well, so that things, they are affecting him, but when he comes to Forest School it will take him out, all these things, he kind of forgets everything after that, yeah…you know, it's changed my life, I would say, yeah, emotionally, because I don't know where I would have been if I don't go for that.. I would have been really in depression or something like that, but it helped me a lot in those days. And after that I just started to take children out more even though it was cold and everything, I just covered them up and I said, "Come on, let's go outside", rather than just sitting at home in front of the telly or doing something, you know, anything, I'll just take them outside and they love it, yeah, yeah, it's lovely.” (Rafi’s mum)*

Making a den to sit in appeared to have a calming effect on the children as a ‘safe space’ where they could chat, play, or just be quiet and take refuge whilst experiencing different emotions. The distraction of the endless natural materials was often irresistible to the children and helped calm the children whose behaviour the nursery staff usually found challenging, or who avoided group activities. Zak occasionally expressed some aggressive behaviours with adults, children and nature during the Forest School sessions. However, when allowed to choose what to do, he would sometimes calm himself down by taking himself off to lie backwards over a fallen tree and stare calmly at the moving branches in the wind.

**Acknowledging and respecting feelings of others:** There were multiple opportunities for children to provide care for each other, adults and wildlife. When walking as a group, Adam was aware of Dalia’s tendency to keep separate from the group and he asked if the staff could check Dalia was still with the group. Tom and Alex were walking together holding hands along a small path, and on approaching a sapling in the way of their path and which Alex hadn’t seen, Tom dragged him over to one side to avoid it.

There were opportunities to enhance their emotional understanding of their peers, for example, the children had been given some soft toys to play with in the den which got packed up for the walk back. When Rafi was allowed to keep one to hold while he walked because he was feeling a bit sad due to his issues at home, some children complained he got a toy when they didn’t. One of the nursery staff, who was holding Rafi’s other hand said that he was feeling a bit sad in that moment and asked they let him carry the toy to make him feel better, which the children accepted. There was also possibly a greater need for children to consider their own wellbeing, as they needed to communicate when they were hungry, tired, needed the toilet, were worried, had had a fall etc. They were also required to consider the adults feelings, such as when Dominic kicked away all the stones from a circle one of the Forest School leaders had made for a pretend fire. He was told that made her (the leader) feel sad, and she asked him to put it back, which he did and was thanked for his cooperation and for considering the feelings of others.

### EYFS 2 Communication and languages

A positive impact on communication and language was observed, through children having opportunities to practice English (their second language) and becoming more verbal in their communication. Examples are provided below.

**Speaking:** Back at the children’s nursery class there was a ratio of 1 staff to 10 children, whereas the Forest School had 4 staff to 10 children. As well as the constantly changing activities, this need to negotiate safety and choice, and expression of what they were experiencing gave children more opportunity and reason to speak to adults and other children. All of the children could speak some English, but many were still learning to speak it in different volumes and would test out adults in their own language to see if they understood. On finding out some didn’t, they struggled on in English. This meant the Forest School was an opportunity to practice English as a second language.

Despite their ability to talk, it is common for children this age to use sounds rather than words, especially with strangers. They may be used to their parents knowing them so well that they often don’t need to use words to communicate their needs. At the beginning of the sessions, Dalia, who the nursery staff described as a disruptive child, used non-verbal communication such as grunts, whines and crying sounds, almost all the time in the first sessions. The Forest School leaders encouraged the children to use words where possible, and Dalia became more cooperative through the course of the programme, using more words:

“ *In nurseries and* schools *, children similar to Dalia are seen as naughty because they need that, they need to explore and they need to be out and express themselves and they need to let off steam and they can’t, and that’s why the beauty of being outside for her was really good*”. *(FS leader 1).*
“ *But she, yeah, I mean she started off didn’t she non-verbal and by the end it was, on that last session Daisy, you know, full* sentence, *‘Daisy look at this mushroom’ or ‘Daisy, Daisy, come over here and look at this’. (FS leader 2)*.

### EYFS 3 Physical development

Through opportunities to be physically active in a larger and more diverse environment than the traditional nursery setting, children demonstrated the ability to run, walk, climb, roll, jump, step and balance.

### EYFS 4 Literacy

There were opportunities to use, expand and refine their vocabulary, when describing their environment, and in order to talk to parents about their new outdoor experiences. Children also corrected each other’s speech. For example, Ali was talking to the evaluator about Maira and described her as ‘him’. Maira shook her head and said she was a ‘her’, after which Ali repeated his sentence with the correct pronoun. There were several instances where children tried to express their feelings and needs where they needed to expand their vocabulary. There were opportunities to talk about synonyms such as Alex saying *“pee pee equals wee wee”* when he saw some bird faeces on a rock. Tom's mum said his vocabulary in his own language was also improving because he would come home having to learn new words in his own language to describe what he had seen. Zak’s dad explained the Forest School helped his son’s vocabulary:

*“But then going to the Forest School, having to, getting in contact with that sort of thing almost every day, getting to know what it is, and I stick it in his head, so it has improved his vocabulary as well, which I'm happy about, because last year we were talking about having help but we've scrapped that now… as well, so yeah, I'm happy about it.”* (Zak’s dad).

### EYFS 5 Mathematics

A positive impact on children’s understanding of mathematical concepts was observed, through opportunities to count, find shapes in the natural environment and discuss the concept of relative size in the natural world. Examples are given below.

**Numbers:** There were multiple opportunities to count things, such as the number of cups needed for everyone to have hot chocolate, cows in the field, round black fungus spots on fallen leaves, children in the group. The attraction of the cows (for many the first cow they had ever seen), lead to a discussion around numbers. Seeing more than three cows required words to describe more than some could count, e.g. “lots”:

*Ali: “there’s 10!”*
*Lina: “No there’s lots of... look, one...two...”*
*Ali: “Alright!”*

Logic and deductive reasoning were challenged when the children were sitting on a narrow path going up a hill, and being asked to pass biscuits down the line until they all had a biscuit. This meant not only did they have to resist eating the biscuit they had to pass on, but they had to understand how every child could get a biscuit, and when to stop passing them on and hold onto their own biscuit.

**Shapes, space and measures:** Relative size was a major theme for the children in a natural environment. Being approximately 3ft tall in the midst of adults, the children were used to seeing themselves as little and being treated as such. When confronted with nature they then found themselves in the position of being big, which some initially disputed. The case of a curled-up slug was such confusion – the children wanted to uncurl a slug they found, so they could see its eyes. The staff said the slug was scared of them because they were like giants to the slug. One boy insisted he wasn’t big and that he was a little boy. The conversation followed as to why the children were big relative to the slug. Another situation where knowledge of relative size was needed, was when Alex wanted his mum to crawl under the fallen tree he had just passed under. He was surprised to see his mum had trouble getting under, and this triggered a conversation between them about their relative size.

Concepts of size were challenged again when Zahra saw a Labrador and a Terrier on a lead, and insisted the Terrier was a baby dog because it was the smallest dog, which provoked another conversation about the logic of size. Other opportunities to apply mathematical concepts were in spatial awareness, for example when spinning around on the log swing and having to ensure they didn’t not kick someone.

Shapes were spotted everywhere, such as the square in the stick den that Zahra pointed out to the adults. The den building was also an opportunity to build 3-dimensional shapes. Discussions about things they saw or found required them to try and use mathematical vocabulary and concepts such as over, under, above, next to, behind, front, back, heavy, light etc. Therefore, the Forest School gave them multiple opportunities to understand and apply mathematical concepts.

### EYFS 6 Understanding the world

The Forest School provided several opportunities to develop experimental skills such as: observing plants and animals; decision making; exploring textures, weights, size, and springiness; and being used to getting hands dirty to facilitate exploring. Through children’s experience, they showed an ability to respect and understand the natural environment. They learned about animal nutrition through watching squirrels find and bury acorns, and through discussing whether leaving a chocolate biscuit would be suitable or eaten if left for any animals. In woods, the life cycle of trees was clear to them when examining seeds and saplings, through to the giants of the 200-year-olds trees and ones that had fallen and were rotting. At first, some of the children were a bit destructive when encountering nature, and tore leaves and sticks off living trees, but soon learnt not to damage living things. It was also an opportunity to understand seasons, as the programme started off when the leaves were golden and red on and off the trees, and by the end had gone black and started to rot. When Maira entered a wood for the first time in her life and saw the brilliant yellow leaves in the sunshine she gasped, gestured and exclaimed “ *Awe, that’s so cute!”.* Dani, who missed three weeks of sessions, saw on his return that the yellow leaves had all fallen off the trees and turned black, and in the pouring rain the tree trunks also looked grim and black. On entering the woods, he exclaimed in disgust “ *Who did this?! Who did this?”.*

### EYFS 7 Expressive arts and design

Using the natural resources available, children were able to use different materials and their imagination to create art and crafts such as making leaf kites; nests; a leaf cake; leaf angel; leaf flag; boot prints; leaf crown and a natural bouquet. Some of these activities were directed by the Forest School leaders, such as the leaf kite; others such as the leaf cake were more spontaneous coming from the children’s ideas. They also demonstrated the ability to participate in imaginative play, such as pretending to be animals and pretending a pile of sticks was a real fire.

## Discussion

This evaluation demonstrated that a pilot Forest School intervention provided developmental benefits for children aged 3 years old in an ethically and socio-economically diverse northern English city. The evaluation successfully captured the voice and experiences of early years children, providing a valuable addition to the current minimal evidence base for this age group. We found the Early Years Foundation Stage profile to be a useful tool to assess the potential benefits of participation in Forest Schools for 3-year-olds. We identified personal, social and emotional development as a particularly important outcomes, but also found benefits for communication and languages; physical development; literacy, mathematics; understanding the world; and expressive arts and design. 

Our evaluation helped to understand the key processes and activities involved in delivering a Forest School for this young age group, and included the child’s voice and experiences to identify developmental outcomes that may have longer term impacts on children’s health and wellbeing. This supports previous findings with similar age groups reported by adult stakeholders associated with Forest Schools ^19^. Forest School exposed children to an increased diversity of materials, activities, environments, problems and opportunities compared to the traditional nursery setting. This, combined with a high staff-to-child ratio, may have contributed to the developmental outcomes observed. Despite the programme only offering 10 sessions, there were examples of potential long-term change, such as Rafi’s mum discovering greenspaces as a way of relieving the impact of her divorce on her and her children; Zahra overcoming her fear of getting her hands messy so she could participate in painting and water play back in class; Zak insisting him and his dad now go to play with nature instead of the playground; and Dalia finding it easier to join in group activities.

Unexpected positive impacts beyond children’s development were observed. Through being inspired to visit green spaces more regularly as a family helped relieve some of the stress of the parents who were going through difficult personal situations. Both parents and children expressed disappointment when the programme finished, and parents who had completed the Forest School training offered had been liaising to visit the woods at weekends and were keen to volunteer at future Forest School programmes at their nursery. Nursery staff were able to enhance their Forest School skills with the support of the Forest School leaders, and discuss ideas they could do with children outside or after the forest school programme. One of the nurseries was developing a Forest School area in their grounds, and had plans to take children off-site in their minibus as a result of developing knowledge of suitable outdoor sites for Forest School activities.

Crucial to the success was the support from existing nursery staff at each session, managing the communication between parents and the project, and having an existing trusted relationship with the parents and children. The much higher staff-to-child ratio in Forest School (4:10) compared to nursery (1:10) was important to ensure the children were comfortable and safe. A high staff-to-child ratio has been previously highlighted as important for supporting children to develop ^33^, however this reduced ratio has cost-implications and could be difficult to fund in additional settings. It will be important for future research to evaluate the effectiveness and cost-effectiveness of this intervention approach. The presence of the Forest School leaders gave the nursery staff a chance to observe the children and work out their behaviours and feelings while they were with the Forest School leaders, helping them to identify and test approaches for dealing with any challenging behaviour.

Although parents were encouraged to engage with Forest School activities, having the parents present was difficult for some of the children as they became clingy and stopped participating in the Forest School, even with their parent’s encouragement. This reduced engagement may have attenuated some of the potential benefits of Forest School.

Our findings are of particular importance for urban settings, where children living in deprived areas may be less likely to be able to access health-promoting, high-quality green spaces in their local environment. The impact of this on children’s health and wellbeing was a significant concern during the Covid-19 pandemic, among children who had previously reported having no access to a garden or nearby park for play, and/or had never played in green spaces of parks ^34^.

Our evaluation had several strengths. Using an ethnographic approach has provided rich data on the child’s experience of the programme (albeit as an interpretation), through participant observation that would have been almost impossible to gain through interviews alone with this young age group; our methods have thus given a voice to young children who may not have otherwise been heard. The data also highlights the children’s large amount of non-verbal communication of feelings, needs, likes and dislikes. Verbal expression included sometimes some quite assertive language:

*“I’m not going, and my coats not going, and my hat’s not going!” [Dominic according to School B Nursery Staff 1]*

However, ethnography outdoors had its challenges, such as note taking in the rain, or malfunctioning backup electronic devices due to cold or rain.

We triangulated observations with other data such as in-depth interviews with parents and staff. We conducted an evaluation with an ethnically diverse group of families living in areas of high deprivation; groups which are often seldom heard. We demonstrated that using the statutory EYFS profile as an outcomes framework was feasible, indicating its potential to be scaled up to evaluate the benefits of Forest School for nursery-age children. This ensures clear policy relevance for education and health providers, given that EYFS data is recorded as part of the nursery education system. This could therefore provide a low-cost opportunity to generate robust evidence on the impact of Forest Schools with nursery-aged children. We also demonstrated wider impact of Forest School engagement among nursery staff and parents, suggesting that outcomes for these Forest School participants should be explored further in future evaluations.

However, there are some weaknesses. We are not able to indicate whether attendance at Forest Schools causally impacted beneficial outcomes. Our sample was small, and located in a deprived, multi-ethnic area and may not be representative of other locations. We recommend further evaluation to explore this using quantitative evaluation using the outcomes identified here. It would also be interesting to repeat the same evaluation with any services set up for even younger children such as the 0–2-year-olds observed in Kemp & Josephidou’s report ^35^ ‘ *Where are the Babies?’.*

The previous play experience of most of the children was almost entirely limited to home, nursery and artificial play equipment only in parks and soft play centres, so had limited to no experience of play in natural and wild spaces. The developmental outcomes observed could therefore have been attributable to the exposure to the new experiences of an outdoor environment, as opposed to the unique Forest School model. A pilot-controlled trial is therefore recommended to explore this further in a similar population.

Given the structural barriers within the built-environment, the findings highlight the need for local policy makers and public health leaders to ensure existing environments are well-managed, maintained and protected from urban development. This is of particular importance in residential areas and near early years educational settings. Planning departments should mandate green space requirements for new residential developments in urban areas that include the creation of high-quality woodland and natural spaces to ensure inequalities aren't exacerbated through lack of access due to the need for transport.

## Conclusion

We found that participation in Forest School for children aged 3 years was beneficial for children’s wellbeing and development, within various domains of the EYFS, particularly in the area of personal, social and emotional development. The ethnographic approach was successfully implemented, and using EYFS as an outcomes framework was feasible. The benefits of Forest School appear to have been influenced by several factors including a higher staff-to-child ratio and diversity of resources and activities than experienced within the early-years setting.

Our findings suggest that engaging in Forest Schools in pre-school settings may have benefits for children to improve their school readiness. Provision of such Forest Schools for this age group may be of particular importance in areas where school readiness levels are low. By increasing the proportion of children who are achieving a good level of development in the EYFS, this could subsequently reduce the need for additional support within the classroom, freeing up teaching support for all children in a class.

## Ethics and consent

This evaluation was deemed by the Health Research Authority to be service evaluation (HRA decision 60/88/81), not research, so ethics approval was not required. Written informed consent for publication of the participants’ details was obtained from the participants/parents of the participant. Children were verbally informed that they would be observed as part of the evaluation.

## Data Availability

Due to rich and highly sensitive data regarding ethnic minorities and very young children we can only share data that is possible to anonymise (interview transcripts and participant characteristics). Observation notes would have to be redacted to ensure confidentiality and photos can only be shared where it is not possible to identify anyone. To request access, please initially contact the authors who hold the data to discuss your request. You will then be required to complete the ‘Expression of Interest’ form available at here and email the BiB research team ( borninbradford@bthft.nhs.uk). If your request is approved, we will ask you to sign a data sharing contract and a data sharing agreement. Harvard Dataverse: Better Start Bradford – Forest School evaluation. https://doi.org/10.7910/DVN/NNFXYE ^36^. This project contains the data collection materials (participant information sheets, consent forms and interview topic guides). Data are available under the terms of the Creative Commons Zero "No rights reserved" data waiver (CC0 1.0 Public domain dedication).
