# Supplementary material for: Unlocking the forest: An ethnographic evaluation of Forest Schools on developmental outcomes for 3-year-olds unaccustomed to woodland spaces
Source: Wellcome Open Res. 2024 Sep 9;9:519. [Version 1] doi: 10.12688/wellcomeopenres.22851.1 (PMC11447439; doi:10.12688/wellcomeopenres.22851.1)
Supplement: Peer review history

# Reviewer report: Reviewer response for version 1

**DOI:** 10.21956/wellcomeopenres.25160.r99157

**Published:** 2024-10-09

**Authors:** Vasiliki Karavida

**Affiliations:** 1 University of Ioannina, Ioannina, Greece

The article "Unlocking the forest: An ethnographic evaluation of Forest Schools on developmental outcomes for 3-year-olds unaccustomed to woodland spaces" is interesting and offers new knowledge on a very interesting topic. It is well-conceived. However, the manuscript can be further improved and strengthened.

Introduction

 The introduction could be more focused on forest schools like the historical review of forest schools. Additional information about the contribution of Forest Schools to child development would enrich the study. On the other hand, the reference to children's play in South Asia is confusing as it seems out of place with the rest of the text.

Methods

- The methods are generally clear.
- However, there is a discrepancy between the number of participants reported in The Intervention and in the Participants, which is confusing. The correct number of participants should be clarified.
- Moreover, there is a discrepancy between the number of sessions in Data collection ( *Participant observation) *and in Discussion.

Discussion

 The discussion mainly repeats the findings of the study. Authors should discuss the results and how they can be interpreted in light of previous studies. In addition, future research directions can also be highlighted.

Is the work clearly and accurately presented and does it cite the current literature?

Yes

If applicable, is the statistical analysis and its interpretation appropriate?

Yes

Are all the source data underlying the results available to ensure full reproducibility?

Yes

Is the study design appropriate and is the work technically sound?

Yes

Are the conclusions drawn adequately supported by the results?

Yes

Are sufficient details of methods and analysis provided to allow replication by others?

Yes

Reviewer Expertise:

My areas of research involve nutrition, healthy lifestyle, education, preschool learning environment and all-round development in early childhood.

I confirm that I have read this submission and believe that I have an appropriate level of expertise to confirm that it is of an acceptable scientific standard.

# Reviewer report: Reviewer response for version 1

**DOI:** 10.21956/wellcomeopenres.25160.r99154

**Published:** 2024-10-02

**Authors:** Avril Johnstone

**Affiliations:** 1 University of Glasgow, Glasgow, UK

Dear Authors,

 I thoroughly enjoyed reading your study, which explores the health and developmental outcomes of participating in a pilot Forest School intervention. This was a comprehensive, novel and interesting piece of research that adds to the limited evidence base. I particularly commend the authors for engaging with seldom heard communities who often have limited experiences of nature.

 I have outlined my feedback and suggestions for further strengthening the article below:

***Is the work clearly and accurately presented and does it engage with the current literature?***

 This is a well written and comprehensive paper that engages effectively with the current literature. However, I believe the introduction could be enhanced by elaborating on the mechanisms through which Forest Schools may benefit children’s physical, social, and emotional development. For example, you could discuss the roles of increased physical activity, social connection, stress reduction, restoration, and the diversification of play (an area that seems underrepresented in your paper). Expanding on these points would help contextualise the results you later present.

***Is the study design appropriate and does the work have academic merit?***

 The Ethnographic approach is an appropriate design choice for a small sample of preschool aged children from mostly ethnic minority backgrounds. This study makes an important contribution as the evidence base for Forest School in preschool aged children is small, particularly research that focuses on ethnically diverse groups from a large Urban area in the UK.

***Are sufficient details of methods and analysis provided to allow replication by others?***

 While the methods are generally clear, there are a few areas where more precision is needed.

 The number of sessions is somewhat confusing. On page 4, you mention “The Forest School provider delivered the programme in four nursery schools over the initial year of delivery (2017–2018), engaging 10 children per nursery.” However, the logic model (Figure 1) mentions 10 x 2-hour sessions, and on page 5, it states that sessions were delivered in various settings (nursery, park, woodland), totalling 11 sessions. Similarly, the number of nurseries involved is inconsistent between pages 4 and 6. Clarifying these details would enhance consistency.

 Related to the above point, I wondered whether the authors felt that this intervention, given that it is only delivered over a short period of time, meets the definition of a Forest School as outlined in the introduction. It might be useful to address this in the paper, acknowledging potential overlaps with other nature-based interventions in early childhood settings.

 I thought the inclusion of the logic model was particularly helpful. Although further development and refinement of this logic model is not the focus of this research, some details could be included. For example, the inputs do not include transport to the woodlands, and the outcomes could have been divided into short and long-term effects to differentiate the likely impacts of participating in the intervention and longer-term effects. Perhaps some of the findings of this study will further refine this logic model. This study by Traynor et al (2022) might be helpful here: "A Low-Cost Method for Understanding How Nature-Based Early Learning and Childcare Impacts Children’s Health and Wellbeing."

 The approach taken for observations and interviews seems appropriate, but I wondered whether observation notes and transcripts were imported into software (e.g., NVivo) to aid analysis. You also mention that “data were partially double coded by a second evaluator.” Could you clarify what this involved and whether it was done to enhance the rigour of the coding process?

***If applicable, is the statistical analysis and its interpretation appropriate?***

 Overall, the analysis seems appropriate. The inclusion of Figures 3 and 4 is particularly useful as descriptions of the nature children are exposed to and what they are engaging in are often lacking in research studies. To aid the interpretation of the results, I thought the integration of a Figure summarising the key findings would be helpful for the reader.

 I was surprised by the limited results related to "EYFS 3 Physical Development."  Given the emphasis on physical play and motor skill development in Forest Schools, I expected more data in this area. Was there a reason for this omission, or was it less emphasized by the participants? Finally, while you mention that both positive and negative impacts would be explored, the results seem predominantly positive. Were there any negative or unintended outcomes reported by staff or parents, and could these be highlighted more explicitly in the results?

***Are all the source data underlying the results available to ensure full reproducibility?***

 This seems appropriate for qualitative data.

***Are the conclusions drawn adequately supported by the results?***

 The conclusions are interesting and align well with the findings presented.

 Overall, this is a strong piece of research, and with the suggested clarifications and expansions, I believe it can make an important contribution to the literature on Forest Schools.

Is the work clearly and accurately presented and does it cite the current literature?

Yes

If applicable, is the statistical analysis and its interpretation appropriate?

Yes

Are all the source data underlying the results available to ensure full reproducibility?

Yes

Is the study design appropriate and is the work technically sound?

Yes

Are the conclusions drawn adequately supported by the results?

Yes

Are sufficient details of methods and analysis provided to allow replication by others?

Partly

Reviewer Expertise:

I am a public health researcher with methodological expertise in developing and evaluating complex interventions and systematic literature reviews. My longstanding research interests focus on exploring the role of active outdoor play, physical activity and/ or nature on reducing inequalities in children's health and wellbeing. Of particular relevance, I led a systematic review on the impacts of nature-based childcare on children's physical, social, emotional and cognitive development.

I confirm that I have read this submission and believe that I have an appropriate level of expertise to confirm that it is of an acceptable scientific standard.

# Reviewer report: Reviewer response for version 1

**DOI:** 10.21956/wellcomeopenres.25160.r99155

**Published:** 2024-09-30

**Authors:** Vahide Yiğit Gençten

**Affiliations:** 1 Pre-school Teaching, Adiyaman University, Adiyaman, Turkey; 2 Pre-school Teaching Department, University of Adiyaman, Adiyaman, Turkey

The article "Unlocking the forest: An ethnographic evaluation of Forest Schools on developmental outcomes for 3-year-olds unaccustomed to woodland spaces" is a well-conceived and valuable contribution to understanding the impact of Forest Schools on young children's development. However, there are several areas where the manuscript can be improved to strengthen its rigor and coherence.

**1. Literature Review:** While the literature review demonstrates familiarity with key studies in the area of early childhood education and outdoor learning, it could benefit from a more structured connection to the broader field of Forest Schools research. The review should better situate the current study within the ongoing scholarly discourse on the benefits of nature-based learning, particularly in relation to young children's social, emotional, and cognitive development. Furthermore, the inclusion of unrelated points, such as the statement regarding "South Asian children’s play in green space" and disparities in access between different ethnic groups, detracts from the focus of the study. Unless these points are directly relevant to the outcomes of Forest Schools or developmental research, they should be removed to maintain clarity and relevance.

**2. Methods Section:** The methods section requires significant restructuring to improve clarity and flow. It would be helpful to organize the section into distinct subsections with clear headings, such as:

- Project Context and Setting
- Participants
- Research Design
- The Intervention
- Data Analysis

This clearer structure will help readers navigate the research process more easily. Additionally, the current version of the methods section should be incorporated within the research design subsection. This is essential for ensuring the study's replicability and transparency.

**3. Ethical Considerations:** There are two separate sections discussing ethical considerations, which creates unnecessary repetition. These sections should be combined into one comprehensive subsection within the methods section. Consolidating the ethical considerations will provide a clear, unified account of how ethical issues were addressed, ensuring the study complies with research standards and best practices.

**4. Inconsistencies in Participant Numbers:** There is a discrepancy between the number of participants mentioned in the abstract and the methods section. This inconsistency must be resolved to avoid confusion. The correct number of participants should be clarified in both sections to maintain accuracy and coherence throughout the manuscript.

**5. Discussion Section:** The discussion section, while insightful, largely reiterates the study's findings without situating them in a broader academic or practical context. To strengthen this section, the authors should explore how their findings contribute to, or challenge, existing theories and practices within Forest Schools or early childhood education. Additionally, including a comparison with findings from similar studies would enrich the discussion and provide a more nuanced understanding of the study's implications.

**6. Conclusion:** Currently, the conclusion mainly summarizes the findings without addressing the study's limitations or practical implications. A more robust conclusion should include a discussion of the limitations of the study, such as sample size, potential biases in data collection, or any constraints in the ethnographic approach. Furthermore, outlining the implications for practitioners, policymakers, or further research would significantly enhance the study's contribution to the field.

 Overall, the article presents valuable insights into the developmental outcomes of 3-year-olds participating in Forest Schools, particularly those unfamiliar with woodland environments. However, revisions are necessary to strengthen the connection to existing literature, improve the clarity of the methods section, resolve inconsistencies, and expand the discussion and conclusion sections to place the findings within a broader context. These revisions will significantly enhance the study's contribution to the field of early childhood education and outdoor learning.

Is the work clearly and accurately presented and does it cite the current literature?

Yes

If applicable, is the statistical analysis and its interpretation appropriate?

Yes

Are all the source data underlying the results available to ensure full reproducibility?

Yes

Is the study design appropriate and is the work technically sound?

Yes

Are the conclusions drawn adequately supported by the results?

Yes

Are sufficient details of methods and analysis provided to allow replication by others?

Yes

Reviewer Expertise:

nature-based learning, early years education, early academic skills

I confirm that I have read this submission and believe that I have an appropriate level of expertise to confirm that it is of an acceptable scientific standard.

## References

[1] : Nature-based reading and writing instructions in early childhood education:The Giving Tree example. *Environmental Education Research* .2022;28(1) : 10.1080/13504622.2021.2015294 95-108 10.1080/13504622.2021.2015294

[2] : The Connection Between Forest School and Preschool Programs: A Comparison Study. *Journal of Qualitative Research in Education* .2023; 10.14689/enad.36.1711 243-260 10.14689/enad.36.1711

[3] : Promoting Environmental Literacy: Nature’s Representation in Kindergarten Books. *European Education* .2024;56(2) : 10.1080/10564934.2024.2355913 126-139 10.1080/10564934.2024.2355913

[4] : NATURE-BASED LEARNING AND DIGITAL COMPETENCIES IN EARLY YEARS EDUCATION. *Nevşehir Hacı Bektaş Veli Üniversitesi SBE Dergisi* .2022;12(4) : 10.30783/nevsosbilen.1123453 2160-2176 10.30783/nevsosbilen.1123453
